# Trisodium bis­{1-[iminio­(morpholino)meth­yl]guanidinium} bis­[hexa­hydrogen­hexa­molybdoaluminate(III)] chloride icosa­hydrate

**DOI:** 10.1107/S1600536808022745

**Published:** 2008-07-26

**Authors:** Feng Wang, Rui-Ge Cao, Yi-Bing Pan, Shu-Xia Liu

**Affiliations:** a Key Laboratory of Polyoxometalates Science of the Ministry of Education, College of Chemistry, Northeast Normal University, Changchun 130024, People’s Republic of China

## Abstract

In the title compound, Na_3_(C_6_H_15_N_5_O)_2_[Al(OH)_6_Mo_6_O_18_]_2_Cl·20H_2_O, the [Al(OH)_6_Mo_6_O_18_]^3−^ polyoxo­anion has a B-type Anderson structure exhibiting approximate *D*_3*d*_ symmetry. There are two types of sodium cations: the Na^+^ cations of type I have a distorted octa­hedral coordination geometry formed by six O atoms and are statistically distributed over two positions with equal occupancies, while the coordination polyhedra of the two Na^+^ cations of type II share one Cl anion located on an inversion center. The latter fragment, containing a Cl anion and two sodium cations, links two polyoxoanions into centrosymmetric blocks. The diprotonated 1-[imino­(morpholino)meth­yl]guanidinium cations and uncordinated water mol­ecules contribute to extensive N—H⋯O and O—H⋯O hydrogen bonding, resulting in the formation a three-dimensional supra­molecular structure.

## Related literature

For related literature, see: Cao *et al.* (2007 ▶); Cheng *et al.* (2007 ▶); Lee *et al.* (1991 ▶); Li *et al.* (2005 ▶); Pope (1983 ▶); Shivaiah *et al.* (2003 ▶); Wang *et al.* (2007 ▶). For general background, see: Brown & Altermatt (1985 ▶).
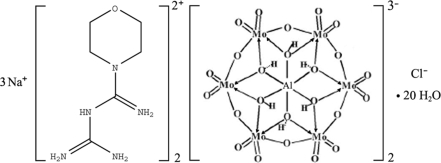


## Experimental

### 

#### Crystal data

Na_3_(C_6_H_15_N_5_O)_2_[Al(OH)_6_Mo_6_O_18_]_2_Cl·20H_2_O
*M* *_r_* = 2796.54
Triclinic, 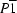

*a* = 10.1070 (6) Å
*b* = 11.3869 (7) Å
*c* = 17.2548 (10) Å
α = 81.6980 (10)°
β = 75.3140 (10)°
γ = 77.0570 (10)°
*V* = 1864.03 (19) Å^3^
*Z* = 1
Mo *K*α radiation
μ = 2.15 mm^−1^
*T* = 296 (2) K
0.31 × 0.25 × 0.22 mm

#### Data collection

Bruker SMART diffractometer
Absorption correction: multi-scan (*SADABS*; Bruker, 1997 ▶) *T*_min_ = 0.528, *T*_max_ = 0.624
9902 measured reflections
6882 independent reflections
6108 reflections with *I* > 2σ(*I*)
*R*_int_ = 0.021

#### Refinement

*R*[*F* ^2^ > 2σ(*F* ^2^)] = 0.034
*wR*(*F* ^2^) = 0.092
*S* = 1.06
6882 reflections
503 parameters
H-atom parameters constrained
Δρ_max_ = 1.36 e Å^−3^
Δρ_min_ = −1.29 e Å^−3^

### 

Data collection: *SMART* (Bruker, 1997 ▶); cell refinement: *SAINT* (Bruker, 1997 ▶); data reduction: *SAINT*; program(s) used to solve structure: *SHELXS97* (Sheldrick, 2008 ▶); program(s) used to refine structure: *SHELXL97* (Sheldrick, 2008 ▶); molecular graphics: *ORTEP-3* (Farrugia, 1997 ▶); software used to prepare material for publication: *SHELXL97*.

## Supplementary Material

Crystal structure: contains datablocks I, global. DOI: 10.1107/S1600536808022745/cv2425sup1.cif

Structure factors: contains datablocks I. DOI: 10.1107/S1600536808022745/cv2425Isup2.hkl

Additional supplementary materials:  crystallographic information; 3D view; checkCIF report

## Figures and Tables

**Table 1:** Hydrogen-bond geometry (Å, °)

*D*—H⋯*A*	*D*—H	H⋯*A*	*D*⋯*A*	*D*—H⋯*A*
N2—H2⋯O8^i^	0.90	2.00	2.855 (5)	158
N3—H3⋯O7^ii^	0.90	1.86	2.750 (5)	173
N5—H6⋯O9^iii^	0.90	1.92	2.821 (5)	179
O1—H8⋯O31	0.85	1.79	2.631 (5)	170
O3—H10⋯O32	0.85	1.87	2.715 (5)	169
O4—H11⋯O27^iv^	0.85	1.90	2.736 (4)	170
O5—H12⋯O30^v^	0.85	1.94	2.769 (5)	164
O6—H13⋯O28^iv^	0.85	1.91	2.737 (4)	164
O26—H14⋯O19^vi^	0.85	2.11	2.919 (5)	159
O26—H15⋯O35^vii^	0.85	2.13	2.898 (5)	150
O27—H16⋯O34^vii^	0.85	2.12	2.918 (5)	155
O27—H17⋯O35^viii^	0.85	1.95	2.784 (5)	169
O28—H18⋯O17^vii^	0.85	2.04	2.845 (5)	158
O28—H19⋯O16^vii^	0.85	1.98	2.790 (5)	160
O29—H20⋯O20^vi^	0.85	2.08	2.853 (5)	152
O29—H21⋯O12^v^	0.84	1.90	2.735 (5)	168
O30—H22⋯O21^vi^	0.85	2.11	2.890 (5)	152
O30—H23⋯O33^v^	0.85	1.93	2.640 (7)	141
O31—H24⋯O32	0.86	2.09	2.889 (7)	154
O31—H25⋯O29	0.86	2.08	2.885 (6)	155
O32—H26⋯O33	0.85	2.00	2.632 (8)	130
O32—H27⋯O23^ix^	0.85	2.03	2.843 (6)	160
O33—H29⋯O10	0.85	1.96	2.812 (6)	176
O34—H30⋯O14^ii^	0.85	2.14	2.991 (5)	175
O34—H31⋯O11^ix^	0.85	1.94	2.756 (5)	161
O35—H32⋯O34	0.85	2.03	2.768 (5)	145
O35—H33⋯O18	0.85	2.14	2.927 (5)	155

Symmetry codes: (i) 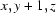; (ii) 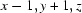; (iii) 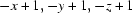; (iv) 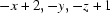; (v) 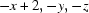; (vi) 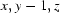; (vii) 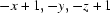; (viii) 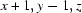; (ix) 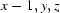.

**Fig. 1.**
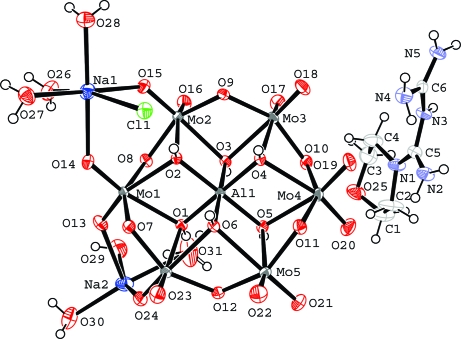
Asymmetric unit of (I), showing 30% probability displacement ellipsoids. Solvent waters have been omitted.

**Fig. 2.**
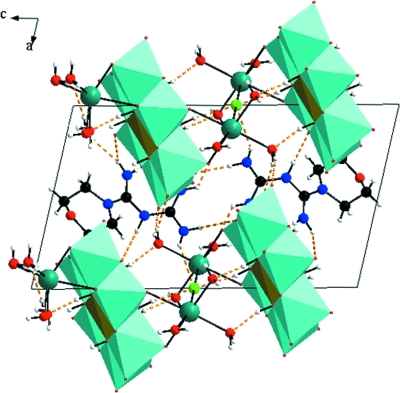
The crystal packing of (I) along b axis with hydrogen bonds drawn as dashed lines. Solvent waters have been omitted.

## supplementary crystallographic information

### Comment

*N*-Amidino-4-morpholincarboxamidine (ABOB) is an effective antiviral agent to influenza, chickenpox, and measles. It is expected that the supramolecular interaction between ABOB and polyoxometalates (POMs) (Pope, 1983) may exhibit synergistic pharmaceutical activity. In our previous work, we have isolated a few compounds based on various polyoxoanions (Cheng *et al.*, 2007; Li *et al.*, 2005; Wang *et al.*, 2007). When investigating the reaction of B-type Anderson polyoxoanion [Al(OH)_6_Mo_6_O_18_]^3^^-^ and ABOB, a compound formulated as Na_3_(H_2_ABOB)_2_[Al(OH)_6_Mo_6_O_18_]_2_Cl^.^20H_2_O (I) was obtained.

The asymmetric unit of (I) is composed of a [Al(OH)_6_Mo_6_O_18_]^3^^-^ polyoxoanion, a protonated ABOB, one and a half Na^+^ cation, half of a Cl^-^ ion, and ten water molecules (Fig. 1). The [Al(OH)_6_Mo_6_O_18_]^3^^-^ polyoxoanion has a B-type Anderson structure in which six {MoO_6_} octahedra arrange hexagonally around the central {Al(OH)_6_} octahedron with approximate *D*_3_*_d_* symmetry; bond lengths and angles are within the normal ranges (Cao *et al*., 2007; Lee *et al.*, 1991; Shivaiah *et al.*, 2003). There are two types - I and II, respectively - of sodium cations: the Na^+^ cations of type I have a distorted octahedral coordination geometry formed by six O atoms and are statistically distributed between two positions with equal occupancies, while coordination polyhedrons of two Na^+^ cations of type II share one Cl anion located on an inversion center. The latter atomic fragment containig a Cl anion and two sodium cations link two polyoxoanions into centrosymmetric blocks. The diprotonated ABOB which acquires two protons from its two imine groups (N2, N4) were adopted to compensate for charge balance. Hydrogen-bonding interactions among the dimer, diprotonated ABOB, water molecules, and Cl^-^ ion result in a three-dimensional supramolecular structure (Fig. 2).

Bond valence sum calculations (Brown & Altermatt, 1985) indicated oxidation states of 5.94–6.01 for the Mo atoms and 2.85 for the Al atom in good agreement with the expected values of 6 and 3, respectively. Furthermore, bond valence sum calculations showed that the oxidation states of the O1–O6 atoms are in the range 1.14–1.22 and the O7–O24 atoms are in the range 1.70–1.91, which agrees with the results of X-ray single-crystal diffraction determination that there are six oxygen atoms protonated.

### Experimental

To a 30 ml aqueous solution of AlCl_3_.6H_2_O (0.36 g, 1.5 mmol), the 10 ml aqueous solution of Na_2_MoO_4_.2H_2_O (0.90 g, 3.7 mmol) and 5 ml of glacial acetic acid were added, respectively, followed by addition of 10 ml ABOB (0.10 g, 0.6 mmol). The pH of the mixture was adjusted with dilute HCl to about 2.6 and it was stirred for half an hour. The filtrate was kept for one week under ambient condition and then block crystals of (I) were collected in about 71% yield based on Mo.

### Refinement

All H atoms (except H24 and H25) were placed in idealized positions and refined as riding model approximation with C–H = 0.97 Å, *U*_iso_(H) = 1.2 *U*_eq_(C), O–H = 0.85 Å, N–H = 0.90 Å and *U*_iso_(H) = 1.2 *U*_eq_(O,*N*). Atoms H24 and H25 (attached to O31) were geometrically positioned and not refined. Na2 atom was treated as disordered over two positions with equal occupancies equal to 0.5.

### Crystal data

**Table:**

Na_3_(C_6_H_15_N_5_O)_2_[Al(OH)_6_Mo_6_O_18_]_2_Cl·20H_2_O	*Z* = 1
*M**_r_* = 2796.54	*F*_000_ = 1364
Triclinic, *P*1	*D*_x_ = 2.491 Mg m^−^^3^
Hall symbol: -P 1	Mo *K*α radiation λ = 0.71073 Å
*a* = 10.1070 (6) Å	Cell parameters from 6444 reflections
*b* = 11.3869 (7) Å	θ = 1.8–25.6º
*c* = 17.2548 (10) Å	µ = 2.15 mm^−^^1^
α = 81.6980 (10)º	*T* = 296 (2) K
β = 75.3140 (10)º	Block, colourless
γ = 77.0570 (10)º	0.31 × 0.25 × 0.22 mm
*V* = 1864.03 (19) Å^3^	

### Data collection

**Table:**

Bruker SMART diffractometer	6882 independent reflections
Radiation source: fine-focus sealed tube	6108 reflections with *I* > 2σ(*I*)
Monochromator: graphite	*R*_int_ = 0.021
Detector resolution: 0 pixels mm^-1^	θ_max_ = 25.6º
*T* = 296(2) K	θ_min_ = 1.8º
ω scans	*h* = −12→12
Absorption correction: multi-scan(SADABS; Bruker, 1997)	*k* = −12→13
*T*_min_ = 0.528, *T*_max_ = 0.624	*l* = −20→20
9902 measured reflections	

### Refinement

**Table:**

Refinement on *F*^2^	Hydrogen site location: inferred from neighbouring sites
Least-squares matrix: full	H-atom parameters constrained
*R*[*F*^2^ > 2σ(*F*^2^)] = 0.034	*w* = 1/[σ^2^(*F*_o_^2^) + (0.0443*P*)^2^ + 4.9534*P*] where *P* = (*F*_o_^2^ + 2*F*_c_^2^)/3
*wR*(*F*^2^) = 0.092	(Δ/σ)_max_ = 0.001
*S* = 1.06	Δρ_max_ = 1.36 e Å^−^^3^
6882 reflections	Δρ_min_ = −1.29 e Å^−^^3^
503 parameters	Extinction correction: SHELXL97 (Sheldrick, 2008), Fc^*^=kFc[1+0.001xFc^2^λ^3^/sin(2θ)]^-1/4^
Primary atom site location: structure-invariant direct methods	Extinction coefficient: 0.0137 (3)
Secondary atom site location: difference Fourier map	

### Special details

**Table:**

Geometry. All e.s.d.'s (except the e.s.d. in the dihedral angle between two l.s. planes) are estimated using the full covariance matrix. The cell e.s.d.'s are taken into account individually in the estimation of e.s.d.'s in distances, angles and torsion angles; correlations between e.s.d.'s in cell parameters are only used when they are defined by crystal symmetry. An approximate (isotropic) treatment of cell e.s.d.'s is used for estimating e.s.d.'s involving l.s. planes.
Refinement. Refinement of *F*^2^ against ALL reflections. The weighted *R*-factor *wR* and goodness of fit *S* are based on *F*^2^, conventional *R*-factors *R* are based on *F*, with *F* set to zero for negative *F*^2^. The threshold expression of *F*^2^ > σ(*F*^2^) is used only for calculating *R*-factors(gt) *etc*. and is not relevant to the choice of reflections for refinement. *R*-factors based on *F*^2^ are statistically about twice as large as those based on *F*, and *R*- factors based on ALL data will be even larger.

### Fractional atomic coordinates and isotropic or equivalent isotropic displacement parameters (Å^2^)

**Table:**

	*x*	*y*	*z*	*U*_iso_*/*U*_eq_	Occ. (<1)
Al1	0.90661 (12)	0.18649 (10)	0.25816 (7)	0.0126 (3)	
Mo1	0.96744 (4)	−0.11386 (3)	0.27426 (2)	0.01484 (11)	
Mo2	0.66462 (4)	0.04088 (3)	0.37024 (2)	0.01508 (11)	
Mo3	0.60225 (4)	0.34140 (3)	0.34933 (2)	0.01652 (11)	
Mo4	0.84344 (4)	0.48692 (3)	0.23681 (2)	0.01781 (11)	
Mo5	1.14688 (4)	0.33344 (3)	0.14622 (2)	0.01812 (11)	
Mo6	1.20687 (4)	0.03452 (3)	0.16141 (2)	0.01669 (11)	
Na1	0.8711 (2)	−0.19733 (17)	0.50224 (11)	0.0280 (4)	
Na2	1.0528 (4)	−0.1445 (4)	0.0459 (2)	0.0327 (9)	0.50
Cl1	1.0000	0.0000	0.5000	0.0242 (3)	
C1	0.4802 (7)	0.6826 (7)	0.0390 (4)	0.0534 (17)	
H1A	0.4184	0.7562	0.0244	0.064*	
H1B	0.5480	0.6580	−0.0096	0.064*	
C2	0.5541 (6)	0.7090 (7)	0.0956 (3)	0.0472 (16)	
H2A	0.5920	0.7812	0.0744	0.057*	
H2B	0.6315	0.6425	0.1002	0.057*	
C3	0.2954 (7)	0.6335 (6)	0.1381 (4)	0.0460 (15)	
H3A	0.2348	0.5752	0.1573	0.055*	
H3B	0.2391	0.7097	0.1222	0.055*	
C4	0.3559 (6)	0.6502 (6)	0.2029 (3)	0.0402 (14)	
H4A	0.3980	0.5716	0.2250	0.048*	
H4B	0.2822	0.6871	0.2453	0.048*	
C5	0.4993 (5)	0.7834 (4)	0.2270 (3)	0.0223 (9)	
C6	0.4182 (5)	0.8042 (4)	0.3722 (3)	0.0197 (9)	
N1	0.4633 (4)	0.7272 (4)	0.1760 (2)	0.0274 (9)	
N2	0.6204 (4)	0.8142 (4)	0.2137 (2)	0.0284 (9)	
H1	0.6948	0.7823	0.1765	0.034*	
H2	0.6464	0.8679	0.2374	0.034*	
N3	0.3989 (4)	0.8116 (3)	0.2962 (2)	0.0214 (8)	
H3	0.3159	0.8429	0.2840	0.026*	
N4	0.5371 (4)	0.7469 (4)	0.3894 (2)	0.0287 (9)	
H4	0.6087	0.7685	0.3512	0.034*	
H5	0.5516	0.7488	0.4385	0.034*	
N5	0.3136 (4)	0.8525 (4)	0.4272 (2)	0.0285 (9)	
H6	0.3317	0.8378	0.4765	0.034*	
H7	0.2377	0.9106	0.4244	0.034*	
O1	0.9705 (3)	0.0596 (3)	0.19008 (17)	0.0151 (6)	
H8	0.9421	0.0643	0.1472	0.018*	
O2	0.8941 (3)	0.0588 (2)	0.34240 (17)	0.0137 (6)	
H9	0.9311	0.0607	0.3812	0.016*	
O3	0.7143 (3)	0.1875 (2)	0.26901 (17)	0.0144 (6)	
H10	0.6828	0.1832	0.2285	0.017*	
O4	0.8404 (3)	0.3137 (3)	0.32567 (17)	0.0151 (6)	
H11	0.8805	0.3146	0.3633	0.018*	
O5	0.9193 (3)	0.3124 (3)	0.17301 (17)	0.0156 (6)	
H12	0.8869	0.3112	0.1323	0.019*	
O6	1.0986 (3)	0.1831 (3)	0.24666 (17)	0.0146 (6)	
H13	1.1312	0.1796	0.2880	0.018*	
O7	1.1509 (3)	−0.0732 (3)	0.25770 (18)	0.0186 (6)	
O8	0.7693 (3)	−0.0652 (3)	0.28711 (18)	0.0181 (6)	
O9	0.6296 (3)	0.1915 (3)	0.41825 (17)	0.0193 (6)	
O10	0.6638 (3)	0.4432 (3)	0.25302 (19)	0.0210 (6)	
O11	1.0414 (3)	0.4388 (3)	0.22920 (19)	0.0211 (7)	
O12	1.1770 (3)	0.1847 (3)	0.09572 (18)	0.0211 (7)	
O13	0.9982 (3)	−0.2022 (3)	0.1980 (2)	0.0272 (7)	
O14	0.9798 (3)	−0.2103 (3)	0.35929 (19)	0.0252 (7)	
O15	0.6820 (3)	−0.0552 (3)	0.45441 (19)	0.0254 (7)	
O16	0.5052 (3)	0.0407 (3)	0.3533 (2)	0.0260 (7)	
O17	0.4406 (3)	0.3449 (3)	0.3343 (2)	0.0291 (8)	
O18	0.5761 (4)	0.4378 (3)	0.4208 (2)	0.0309 (8)	
O19	0.8044 (4)	0.5837 (3)	0.3101 (2)	0.0292 (8)	
O20	0.8410 (4)	0.5760 (3)	0.1485 (2)	0.0333 (8)	
O21	1.1373 (4)	0.4294 (3)	0.0617 (2)	0.0332 (8)	
O22	1.3079 (4)	0.3260 (3)	0.1628 (2)	0.0328 (8)	
O23	1.3700 (3)	0.0303 (3)	0.1751 (2)	0.0293 (8)	
O24	1.2263 (4)	−0.0587 (3)	0.08914 (19)	0.0282 (7)	
O25	0.4015 (4)	0.5917 (4)	0.0695 (2)	0.0443 (10)	
O26	0.7727 (4)	−0.3471 (3)	0.4712 (2)	0.0412 (9)	
H14	0.7606	−0.3567	0.4257	0.049*	
H15	0.7767	−0.4063	0.5072	0.049*	
O27	1.0513 (4)	−0.3434 (3)	0.5500 (2)	0.0320 (8)	
H16	1.0269	−0.4106	0.5687	0.038*	
H17	1.1231	−0.3797	0.5185	0.038*	
O28	0.7555 (3)	−0.1911 (3)	0.6411 (2)	0.0287 (7)	
H18	0.7086	−0.2468	0.6578	0.034*	
H19	0.6784	−0.1420	0.6539	0.034*	
O29	0.8521 (5)	−0.1943 (4)	0.0585 (2)	0.0469 (10)	
H20	0.8663	−0.2705	0.0695	0.056*	
H21	0.8414	−0.2015	0.0126	0.056*	
O30	1.1468 (5)	−0.3344 (4)	−0.0247 (3)	0.0468 (10)	
H22	1.1144	−0.3929	0.0046	0.056*	
H23	1.2350	−0.3517	−0.0313	0.056*	
O31	0.8656 (6)	0.0541 (4)	0.0661 (3)	0.0680 (16)	
H24	0.7963	0.1050	0.0906	0.082*	
H25	0.8343	−0.0111	0.0688	0.082*	
O32	0.5896 (5)	0.1589 (5)	0.1528 (3)	0.0566 (12)	
H26	0.5532	0.2243	0.1292	0.068*	
H27	0.5356	0.1083	0.1671	0.068*	
O33	0.5983 (6)	0.3897 (5)	0.1147 (3)	0.0774 (16)	
H28	0.5206	0.4255	0.1042	0.093*	
H29	0.6148	0.4084	0.1568	0.093*	
O34	0.1012 (4)	0.5252 (3)	0.3550 (2)	0.0393 (9)	
H31	0.1008	0.4880	0.3158	0.047*	
H30	0.0624	0.5995	0.3560	0.047*	
O35	0.2881 (4)	0.5691 (4)	0.4350 (2)	0.0400 (9)	
H32	0.2634	0.5508	0.3954	0.048*	
H33	0.3707	0.5461	0.4418	0.048*	

### Atomic displacement parameters (Å^2^)

**Table:**

	*U*^11^	*U*^22^	*U*^33^	*U*^12^	*U*^13^	*U*^23^
Al1	0.0114 (6)	0.0125 (6)	0.0131 (6)	0.0000 (5)	−0.0035 (5)	−0.0004 (5)
Mo1	0.01445 (19)	0.01288 (19)	0.01581 (19)	−0.00011 (14)	−0.00296 (14)	−0.00181 (14)
Mo2	0.01243 (19)	0.01500 (19)	0.01653 (19)	−0.00142 (14)	−0.00272 (14)	−0.00036 (14)
Mo3	0.0136 (2)	0.01542 (19)	0.0182 (2)	0.00193 (14)	−0.00290 (14)	−0.00296 (14)
Mo4	0.0185 (2)	0.01218 (19)	0.0213 (2)	0.00010 (14)	−0.00548 (15)	0.00012 (14)
Mo5	0.0164 (2)	0.0168 (2)	0.0192 (2)	−0.00425 (14)	−0.00140 (15)	0.00134 (15)
Mo6	0.0132 (2)	0.01648 (19)	0.0180 (2)	0.00022 (14)	−0.00215 (14)	−0.00154 (14)
Na1	0.0300 (10)	0.0288 (10)	0.0256 (10)	−0.0043 (8)	−0.0102 (8)	0.0007 (8)
Na2	0.034 (2)	0.040 (2)	0.026 (2)	−0.0118 (18)	−0.0036 (17)	−0.0084 (17)
Cl1	0.0264 (8)	0.0258 (8)	0.0232 (8)	−0.0042 (6)	−0.0117 (6)	−0.0015 (6)
C1	0.053 (4)	0.076 (5)	0.033 (3)	−0.020 (3)	0.001 (3)	−0.018 (3)
C2	0.034 (3)	0.086 (5)	0.026 (3)	−0.025 (3)	0.010 (2)	−0.025 (3)
C3	0.043 (3)	0.047 (4)	0.053 (4)	−0.018 (3)	−0.006 (3)	−0.014 (3)
C4	0.034 (3)	0.053 (4)	0.036 (3)	−0.022 (3)	0.005 (2)	−0.016 (3)
C5	0.021 (2)	0.026 (2)	0.019 (2)	0.0008 (18)	−0.0077 (18)	−0.0025 (18)
C6	0.025 (2)	0.015 (2)	0.020 (2)	−0.0021 (17)	−0.0081 (18)	−0.0012 (17)
N1	0.025 (2)	0.041 (2)	0.019 (2)	−0.0114 (18)	−0.0028 (16)	−0.0093 (18)
N2	0.022 (2)	0.038 (2)	0.027 (2)	−0.0077 (18)	−0.0031 (17)	−0.0125 (18)
N3	0.0178 (19)	0.029 (2)	0.0152 (18)	0.0043 (15)	−0.0063 (15)	−0.0052 (15)
N4	0.027 (2)	0.035 (2)	0.025 (2)	−0.0001 (18)	−0.0141 (17)	0.0013 (18)
N5	0.030 (2)	0.031 (2)	0.022 (2)	0.0012 (18)	−0.0032 (17)	−0.0094 (17)
O1	0.0162 (15)	0.0164 (14)	0.0132 (14)	−0.0020 (11)	−0.0045 (11)	−0.0027 (11)
O2	0.0129 (14)	0.0144 (14)	0.0142 (14)	−0.0005 (11)	−0.0067 (11)	0.0007 (11)
O3	0.0119 (14)	0.0165 (14)	0.0155 (14)	−0.0002 (11)	−0.0064 (11)	−0.0018 (11)
O4	0.0145 (15)	0.0146 (14)	0.0179 (14)	−0.0011 (11)	−0.0074 (11)	−0.0033 (11)
O5	0.0167 (15)	0.0171 (14)	0.0133 (14)	−0.0006 (11)	−0.0080 (11)	0.0017 (11)
O6	0.0115 (14)	0.0164 (14)	0.0166 (14)	−0.0014 (11)	−0.0051 (11)	−0.0025 (11)
O7	0.0129 (15)	0.0193 (15)	0.0225 (16)	−0.0011 (12)	−0.0055 (12)	0.0011 (12)
O8	0.0141 (15)	0.0182 (15)	0.0235 (16)	−0.0022 (12)	−0.0059 (12)	−0.0057 (12)
O9	0.0199 (16)	0.0187 (15)	0.0158 (15)	0.0000 (12)	−0.0006 (12)	−0.0029 (12)
O10	0.0157 (15)	0.0183 (15)	0.0269 (17)	0.0015 (12)	−0.0079 (13)	0.0026 (13)
O11	0.0159 (15)	0.0197 (15)	0.0281 (17)	−0.0025 (12)	−0.0059 (13)	−0.0041 (13)
O12	0.0244 (17)	0.0192 (15)	0.0160 (15)	−0.0022 (13)	−0.0007 (12)	0.0000 (12)
O13	0.0271 (18)	0.0250 (17)	0.0298 (18)	0.0005 (14)	−0.0063 (14)	−0.0129 (14)
O14	0.0274 (18)	0.0184 (16)	0.0260 (17)	−0.0006 (13)	−0.0060 (14)	0.0038 (13)
O15	0.0261 (18)	0.0222 (16)	0.0238 (17)	−0.0019 (13)	−0.0040 (14)	0.0043 (13)
O16	0.0163 (16)	0.0260 (17)	0.0361 (19)	−0.0034 (13)	−0.0073 (14)	−0.0027 (14)
O17	0.0187 (17)	0.0305 (18)	0.0363 (19)	−0.0011 (14)	−0.0097 (14)	0.0026 (15)
O18	0.033 (2)	0.0261 (18)	0.0303 (19)	0.0022 (15)	−0.0020 (15)	−0.0124 (15)
O19	0.0308 (19)	0.0184 (16)	0.039 (2)	−0.0014 (14)	−0.0072 (16)	−0.0097 (14)
O20	0.034 (2)	0.0271 (18)	0.035 (2)	−0.0041 (15)	−0.0101 (16)	0.0115 (15)
O21	0.041 (2)	0.0246 (18)	0.0289 (18)	−0.0069 (15)	−0.0048 (16)	0.0087 (15)
O22	0.0211 (18)	0.0329 (19)	0.046 (2)	−0.0099 (15)	−0.0063 (16)	−0.0031 (17)
O23	0.0155 (16)	0.0285 (18)	0.043 (2)	−0.0030 (13)	−0.0106 (15)	0.0038 (15)
O24	0.035 (2)	0.0224 (17)	0.0234 (17)	−0.0013 (14)	−0.0014 (14)	−0.0051 (14)
O25	0.049 (3)	0.049 (2)	0.037 (2)	−0.015 (2)	0.0010 (18)	−0.0233 (19)
O26	0.056 (3)	0.035 (2)	0.037 (2)	−0.0177 (19)	−0.0168 (19)	0.0055 (17)
O27	0.0309 (19)	0.0327 (19)	0.0318 (19)	0.0024 (15)	−0.0117 (15)	−0.0068 (15)
O28	0.0192 (17)	0.038 (2)	0.0297 (18)	−0.0064 (14)	−0.0061 (14)	−0.0035 (15)
O29	0.061 (3)	0.047 (2)	0.031 (2)	0.003 (2)	−0.0201 (19)	−0.0056 (18)
O30	0.057 (3)	0.035 (2)	0.053 (3)	−0.0123 (19)	−0.025 (2)	0.0108 (19)
O31	0.107 (4)	0.041 (2)	0.076 (3)	0.010 (3)	−0.070 (3)	−0.020 (2)
O32	0.049 (3)	0.078 (3)	0.050 (3)	−0.016 (2)	−0.024 (2)	0.002 (2)
O33	0.068 (4)	0.094 (4)	0.075 (4)	−0.002 (3)	−0.029 (3)	−0.021 (3)
O34	0.038 (2)	0.036 (2)	0.048 (2)	−0.0004 (17)	−0.0182 (18)	−0.0154 (18)
O35	0.029 (2)	0.044 (2)	0.048 (2)	0.0031 (17)	−0.0107 (17)	−0.0188 (19)

### Geometric parameters (Å, °)

**Table:**

Al1—O4	1.887 (3)	Na2—H25	2.3601
Al1—O1	1.890 (3)	Cl1—Na1^ii^	2.8298 (19)
Al1—O6	1.892 (3)	C1—O25	1.408 (7)
Al1—O5	1.900 (3)	C1—C2	1.468 (8)
Al1—O3	1.902 (3)	C1—H1A	0.9700
Al1—O2	1.903 (3)	C1—H1B	0.9700
Mo1—O13	1.696 (3)	C2—N1	1.471 (6)
Mo1—O14	1.715 (3)	C2—H2A	0.9700
Mo1—O8	1.918 (3)	C2—H2B	0.9700
Mo1—O7	1.953 (3)	C3—O25	1.439 (7)
Mo1—O1	2.277 (3)	C3—C4	1.455 (8)
Mo1—O2	2.317 (3)	C3—H3A	0.9700
Mo2—O15	1.709 (3)	C3—H3B	0.9700
Mo2—O16	1.709 (3)	C4—N1	1.488 (6)
Mo2—O9	1.934 (3)	C4—H4A	0.9700
Mo2—O8	1.948 (3)	C4—H4B	0.9700
Mo2—O3	2.276 (3)	C5—N2	1.304 (6)
Mo2—O2	2.296 (3)	C5—N1	1.321 (6)
Mo3—O18	1.700 (3)	C5—N3	1.383 (6)
Mo3—O17	1.709 (3)	C6—N5	1.304 (6)
Mo3—O10	1.932 (3)	C6—N4	1.316 (6)
Mo3—O9	1.943 (3)	C6—N3	1.361 (5)
Mo3—O4	2.293 (3)	N2—H1	0.9000
Mo3—O3	2.300 (3)	N2—H2	0.8998
Mo4—O20	1.706 (3)	N3—H3	0.8999
Mo4—O19	1.710 (3)	N4—H4	0.9000
Mo4—O11	1.928 (3)	N4—H5	0.9000
Mo4—O10	1.931 (3)	N5—H6	0.8999
Mo4—O5	2.296 (3)	N5—H7	0.8999
Mo4—O4	2.318 (3)	O1—H8	0.8500
Mo5—O21	1.701 (3)	O2—H9	0.8500
Mo5—O22	1.704 (3)	O3—H10	0.8501
Mo5—O11	1.943 (3)	O4—H11	0.8499
Mo5—O12	1.945 (3)	O5—H12	0.8500
Mo5—O5	2.288 (3)	O6—H13	0.8499
Mo5—O6	2.293 (3)	O26—H14	0.8499
Mo6—O24	1.698 (3)	O26—H15	0.8501
Mo6—O23	1.713 (3)	O27—H16	0.8500
Mo6—O12	1.919 (3)	O27—H17	0.8500
Mo6—O7	1.957 (3)	O28—H18	0.8500
Mo6—O1	2.274 (3)	O28—H19	0.8500
Mo6—O6	2.301 (3)	O29—H20	0.8486
Na1—O26	2.340 (4)	O29—H21	0.8441
Na1—O28	2.388 (4)	O30—H22	0.8500
Na1—O27	2.402 (4)	O30—H23	0.8501
Na1—O14	2.444 (4)	O31—Na2^i^	2.116 (7)
Na1—O15	2.446 (4)	O31—H24	0.8624
Na1—Cl1	2.8298 (19)	O31—H25	0.8617
Na2—O31^i^	2.116 (7)	O32—H26	0.8501
Na2—O29	2.177 (6)	O32—H27	0.8500
Na2—O24	2.496 (5)	O33—H28	0.8500
Na2—O30	2.513 (6)	O33—H29	0.8500
Na2—O13	2.559 (5)	O34—H31	0.8499
Na2—O31	2.607 (6)	O34—H30	0.8500
Na2—Na2^i^	3.502 (9)	O35—H32	0.8500
Na2—H20	2.5362	O35—H33	0.8500
Na2—H21	2.5822		
			
O4—Al1—O1	179.20 (13)	O29—Na2—H20	18.8
O4—Al1—O6	96.29 (13)	O24—Na2—H20	153.3
O1—Al1—O6	84.45 (13)	O30—Na2—H20	69.5
O4—Al1—O5	84.77 (13)	O13—Na2—H20	75.6
O1—Al1—O5	94.99 (13)	O31—Na2—H20	91.6
O6—Al1—O5	84.48 (13)	Na2^i^—Na2—H20	111.3
O4—Al1—O3	84.60 (13)	O31^i^—Na2—H21	99.6
O1—Al1—O3	94.66 (13)	O29—Na2—H21	18.0
O6—Al1—O3	179.09 (13)	O24—Na2—H21	169.8
O5—Al1—O3	95.81 (13)	O30—Na2—H21	74.0
O4—Al1—O2	96.00 (13)	O13—Na2—H21	99.1
O1—Al1—O2	84.23 (13)	O31—Na2—H21	77.7
O6—Al1—O2	95.63 (13)	Na2^i^—Na2—H21	86.7
O5—Al1—O2	179.20 (13)	H20—Na2—H21	27.3
O3—Al1—O2	84.06 (13)	O31^i^—Na2—H25	95.2
O13—Mo1—O14	106.44 (16)	O29—Na2—H25	54.5
O13—Mo1—O8	97.66 (14)	O24—Na2—H25	109.9
O14—Mo1—O8	101.93 (14)	O30—Na2—H25	133.5
O13—Mo1—O7	100.20 (15)	O13—Na2—H25	86.1
O14—Mo1—O7	95.41 (14)	O31—Na2—H25	19.2
O8—Mo1—O7	150.41 (12)	Na2^i^—Na2—H25	49.6
O13—Mo1—O1	93.31 (14)	H20—Na2—H25	72.4
O14—Mo1—O1	158.53 (13)	H21—Na2—H25	60.0
O8—Mo1—O1	83.25 (12)	Na1—Cl1—Na1^ii^	180.00 (4)
O7—Mo1—O1	72.38 (11)	O25—C1—C2	114.0 (5)
O13—Mo1—O2	158.48 (14)	O25—C1—H1A	108.8
O14—Mo1—O2	94.31 (13)	C2—C1—H1A	108.8
O8—Mo1—O2	71.94 (11)	O25—C1—H1B	108.8
O7—Mo1—O2	83.10 (11)	C2—C1—H1B	108.8
O1—Mo1—O2	67.22 (10)	H1A—C1—H1B	107.7
O15—Mo2—O16	107.36 (16)	C1—C2—N1	112.6 (5)
O15—Mo2—O9	97.76 (14)	C1—C2—H2A	109.1
O16—Mo2—O9	101.84 (14)	N1—C2—H2A	109.1
O15—Mo2—O8	100.08 (14)	C1—C2—H2B	109.1
O16—Mo2—O8	94.96 (14)	N1—C2—H2B	109.1
O9—Mo2—O8	150.56 (12)	H2A—C2—H2B	107.8
O15—Mo2—O3	157.71 (14)	O25—C3—C4	111.5 (5)
O16—Mo2—O3	94.40 (13)	O25—C3—H3A	109.3
O9—Mo2—O3	72.44 (11)	C4—C3—H3A	109.3
O8—Mo2—O3	82.39 (12)	O25—C3—H3B	109.3
O15—Mo2—O2	91.79 (13)	C4—C3—H3B	109.3
O16—Mo2—O2	158.68 (13)	H3A—C3—H3B	108.0
O9—Mo2—O2	84.31 (12)	C3—C4—N1	112.9 (5)
O8—Mo2—O2	71.95 (11)	C3—C4—H4A	109.0
O3—Mo2—O2	67.73 (10)	N1—C4—H4A	109.0
O18—Mo3—O17	105.49 (17)	C3—C4—H4B	109.0
O18—Mo3—O10	100.65 (15)	N1—C4—H4B	109.0
O17—Mo3—O10	96.30 (15)	H4A—C4—H4B	107.8
O18—Mo3—O9	97.33 (15)	N2—C5—N1	123.4 (4)
O17—Mo3—O9	101.13 (15)	N2—C5—N3	120.5 (4)
O10—Mo3—O9	150.56 (12)	N1—C5—N3	116.0 (4)
O18—Mo3—O4	93.17 (14)	N5—C6—N4	121.7 (4)
O17—Mo3—O4	159.96 (14)	N5—C6—N3	117.6 (4)
O10—Mo3—O4	72.67 (11)	N4—C6—N3	120.7 (4)
O9—Mo3—O4	83.28 (12)	C5—N1—C2	120.3 (4)
O18—Mo3—O3	158.31 (14)	C5—N1—C4	122.1 (4)
O17—Mo3—O3	95.15 (14)	C2—N1—C4	115.3 (4)
O10—Mo3—O3	83.29 (12)	C5—N2—H1	122.8
O9—Mo3—O3	71.74 (11)	C5—N2—H2	129.3
O4—Mo3—O3	67.44 (10)	H1—N2—H2	107.9
O20—Mo4—O19	106.06 (17)	C6—N3—C5	127.3 (4)
O20—Mo4—O11	100.82 (16)	C6—N3—H3	122.6
O19—Mo4—O11	98.43 (15)	C5—N3—H3	109.9
O20—Mo4—O10	97.79 (15)	C6—N4—H4	109.8
O19—Mo4—O10	99.41 (15)	C6—N4—H5	119.8
O11—Mo4—O10	149.46 (13)	H4—N4—H5	109.9
O20—Mo4—O5	92.72 (14)	C6—N5—H6	112.3
O19—Mo4—O5	160.56 (14)	C6—N5—H7	132.5
O11—Mo4—O5	72.58 (11)	H6—N5—H7	113.4
O10—Mo4—O5	82.59 (12)	Al1—O1—Mo6	104.56 (13)
O20—Mo4—O4	158.16 (14)	Al1—O1—Mo1	105.23 (13)
O19—Mo4—O4	94.86 (14)	Mo6—O1—Mo1	93.54 (11)
O11—Mo4—O4	81.85 (12)	Al1—O1—H8	120.5
O10—Mo4—O4	72.12 (11)	Mo6—O1—H8	110.4
O5—Mo4—O4	67.18 (10)	Mo1—O1—H8	118.5
O21—Mo5—O22	106.29 (18)	Al1—O2—Mo2	103.71 (12)
O21—Mo5—O11	100.95 (15)	Al1—O2—Mo1	103.31 (12)
O22—Mo5—O11	97.37 (15)	Mo2—O2—Mo1	92.32 (10)
O21—Mo5—O12	96.44 (15)	Al1—O2—H9	117.7
O22—Mo5—O12	100.96 (15)	Mo2—O2—H9	119.0
O11—Mo5—O12	150.02 (13)	Mo1—O2—H9	116.9
O21—Mo5—O5	94.55 (15)	Al1—O3—Mo2	104.49 (12)
O22—Mo5—O5	158.37 (14)	Al1—O3—Mo3	103.59 (12)
O11—Mo5—O5	72.51 (11)	Mo2—O3—Mo3	93.06 (10)
O12—Mo5—O5	81.98 (12)	Al1—O3—H10	121.1
O21—Mo5—O6	159.79 (14)	Mo2—O3—H10	112.8
O22—Mo5—O6	92.62 (14)	Mo3—O3—H10	117.6
O11—Mo5—O6	83.22 (12)	Al1—O4—Mo3	104.35 (13)
O12—Mo5—O6	72.48 (11)	Al1—O4—Mo4	103.82 (13)
O5—Mo5—O6	67.61 (10)	Mo3—O4—Mo4	91.65 (10)
O24—Mo6—O23	107.00 (17)	Al1—O4—H11	119.0
O24—Mo6—O12	97.20 (14)	Mo3—O4—H11	121.2
O23—Mo6—O12	101.23 (15)	Mo4—O4—H11	112.5
O24—Mo6—O7	99.93 (14)	Al1—O5—Mo5	103.92 (12)
O23—Mo6—O7	95.46 (15)	Al1—O5—Mo4	104.23 (13)
O12—Mo6—O7	151.39 (12)	Mo5—O5—Mo4	91.93 (10)
O24—Mo6—O1	92.32 (14)	Al1—O5—H12	120.6
O23—Mo6—O1	158.90 (14)	Mo5—O5—H12	116.0
O12—Mo6—O1	84.23 (12)	Mo4—O5—H12	115.8
O7—Mo6—O1	72.38 (11)	Al1—O6—Mo5	103.97 (13)
O24—Mo6—O6	157.89 (14)	Al1—O6—Mo6	103.45 (12)
O23—Mo6—O6	94.42 (14)	Mo5—O6—Mo6	91.97 (10)
O12—Mo6—O6	72.74 (11)	Al1—O6—H13	120.2
O7—Mo6—O6	83.02 (11)	Mo5—O6—H13	117.2
O1—Mo6—O6	67.51 (10)	Mo6—O6—H13	115.6
O26—Na1—O28	98.76 (15)	Mo1—O7—Mo6	116.00 (15)
O26—Na1—O27	92.75 (15)	Mo1—O8—Mo2	118.76 (15)
O28—Na1—O27	85.13 (13)	Mo2—O9—Mo3	117.88 (15)
O26—Na1—O14	78.03 (14)	Mo4—O10—Mo3	117.74 (15)
O28—Na1—O14	176.70 (14)	Mo4—O11—Mo5	116.73 (15)
O27—Na1—O14	95.76 (13)	Mo6—O12—Mo5	117.58 (15)
O26—Na1—O15	85.00 (14)	Mo1—O13—Na2	129.4 (2)
O28—Na1—O15	94.41 (13)	Mo1—O14—Na1	133.86 (17)
O27—Na1—O15	177.61 (15)	Mo2—O15—Na1	136.74 (18)
O14—Na1—O15	84.57 (12)	Mo6—O24—Na2	131.4 (2)
O26—Na1—Cl1	166.36 (12)	C1—O25—C3	107.9 (4)
O28—Na1—Cl1	93.76 (10)	Na1—O26—H14	127.7
O27—Na1—Cl1	93.69 (11)	Na1—O26—H15	109.1
O14—Na1—Cl1	89.35 (10)	H14—O26—H15	120.0
O15—Na1—Cl1	88.69 (10)	Na1—O27—H16	113.0
O31^i^—Na2—O29	116.0 (2)	Na1—O27—H17	122.8
O31^i^—Na2—O24	82.05 (19)	H16—O27—H17	90.5
O29—Na2—O24	155.2 (2)	Na1—O28—H18	112.3
O31^i^—Na2—O30	85.3 (2)	Na1—O28—H19	118.8
O29—Na2—O30	83.64 (19)	H18—O28—H19	86.1
O24—Na2—O30	116.1 (2)	Na2—O29—H20	105.3
O31^i^—Na2—O13	159.2 (2)	Na2—O29—H21	109.3
O29—Na2—O13	81.59 (18)	H20—O29—H21	91.0
O24—Na2—O13	77.99 (15)	Na2—O30—H22	109.5
O30—Na2—O13	108.83 (19)	Na2—O30—H23	109.4
O31^i^—Na2—O31	84.8 (3)	H22—O30—H23	107.5
O29—Na2—O31	73.6 (2)	Na2^i^—O31—Na2	95.2 (3)
O24—Na2—O31	92.50 (18)	Na2^i^—O31—H24	106.6
O30—Na2—O31	148.0 (2)	Na2—O31—H24	158.0
O13—Na2—O31	90.10 (18)	Na2^i^—O31—H25	121.3
O31^i^—Na2—Na2^i^	47.84 (16)	Na2—O31—H25	64.0
O29—Na2—Na2^i^	93.1 (2)	H24—O31—H25	105.3
O24—Na2—Na2^i^	87.08 (17)	H26—O32—H27	112.7
O30—Na2—Na2^i^	125.6 (2)	H28—O33—H29	115.7
O13—Na2—Na2^i^	124.5 (2)	H31—O34—H30	118.8
O31—Na2—Na2^i^	36.99 (15)	H32—O35—H33	122.5
O31^i^—Na2—H20	124.6		

Symmetry codes: (i) −*x*+2, −*y*, −*z*; (ii) −*x*+2, −*y*, −*z*+1.

### Hydrogen-bond geometry (Å, °)

**Table:**

*D*—H···*A*	*D*—H	H···*A*	*D*···*A*	*D*—H···*A*
N2—H2···O8^iii^	0.90	2.00	2.855 (5)	158
N3—H3···O7^iv^	0.90	1.86	2.750 (5)	173
N5—H6···O9^v^	0.90	1.92	2.821 (5)	179
O1—H8···O31	0.85	1.79	2.631 (5)	170
O3—H10···O32	0.85	1.87	2.715 (5)	169
O4—H11···O27^ii^	0.85	1.90	2.736 (4)	170
O5—H12···O30^i^	0.85	1.94	2.769 (5)	164
O6—H13···O28^ii^	0.85	1.91	2.737 (4)	164
O26—H14···O19^vi^	0.85	2.11	2.919 (5)	159
O26—H15···O35^vii^	0.85	2.13	2.898 (5)	150
O27—H16···O34^vii^	0.85	2.12	2.918 (5)	155
O27—H17···O35^viii^	0.85	1.95	2.784 (5)	169
O28—H18···O17^vii^	0.85	2.04	2.845 (5)	158
O28—H19···O16^vii^	0.85	1.98	2.790 (5)	160
O29—H20···O20^vi^	0.85	2.08	2.853 (5)	152
O29—H21···O12^i^	0.84	1.90	2.735 (5)	168
O30—H22···O21^vi^	0.85	2.11	2.890 (5)	152
O30—H23···O33^i^	0.85	1.93	2.640 (7)	141
O31—H24···O32	0.86	2.09	2.889 (7)	154
O31—H25···O29	0.86	2.08	2.885 (6)	155
O32—H26···O33	0.85	2.00	2.632 (8)	130
O32—H27···O23^ix^	0.85	2.03	2.843 (6)	160
O33—H29···O10	0.85	1.96	2.812 (6)	176
O34—H30···O14^iv^	0.85	2.14	2.991 (5)	175
O34—H31···O11^ix^	0.85	1.94	2.756 (5)	161
O35—H32···O34	0.85	2.03	2.768 (5)	145
O35—H33···O18	0.85	2.14	2.927 (5)	155

Symmetry codes: (iii) *x*, *y*+1, *z*; (iv) *x*−1, *y*+1, *z*; (v) −*x*+1, −*y*+1, −*z*+1; (ii) −*x*+2, −*y*, −*z*+1; (i) −*x*+2, −*y*, −*z*; (vi) *x*, *y*−1, *z*; (vii) −*x*+1, −*y*, −*z*+1; (viii) *x*+1, *y*−1, *z*; (ix) *x*−1, *y*, *z*.

## References

[bb1] Brown, I. D. & Altermatt, D. (1985). *Acta Cryst.* B**41**, 244–247.

[bb2] Bruker (1997). *SMART*, *SAINT* and *SADABS* Bruker AXS Inc., Madison, Wisconsin, USA.

[bb3] Cao, R. G., Liu, S. X., Xie, L. H., Pan, Y. B., Cao, J. F., Ren, Y. H. & Xu, L. (2007). *Inorg* *Chem* **46**, 3541–3547. 10.1021/ic062208c 17408263

[bb4] Cheng, H. Y., Liu, S. X., Xie, L. H., Ren, Y. H. & Zhang, C. D. (2007). *Chem* *Lett* **36**, 746–747.

[bb5] Farrugia, L. J. (1997). *J* *Appl* *Cryst* **30**, 565.

[bb6] Lee, H. Y., Park, K. M., Lee, U. & Ichida, H. (1991). *Acta Cryst.* C**47**, 1959–1961.

[bb7] Li, D. H., Liu, S. X., Sun, C. Y., Xie, L. H., Wang, E. B., Hu, N. H. & Jia, H. Q. (2005). *Inorg* *Chem* *Commun* **8**, 433–436.

[bb8] Pope, M. T. (1983). *Heteropoly and Isopoly Oxometalates* New York: Springer-Verlag.

[bb9] Sheldrick, G. M. (2008). *Acta Cryst.* A**64**, 112–122. 10.1107/S0108767307043930 18156677

[bb10] Shivaiah, V., Nagaraju, M. & Das, S. K. (2003). *Inorg* *Chem* **42**, 6604–6606. 10.1021/ic034581f 14552610

[bb11] Wang, F., Liu, S.-X., Wang, C.-L., Cao, R.-G. & Cao, J.-F. (2007). *Acta Cryst.* E**63**, m1708–m1709.

